# Intercropping of Stylosanthes green manure could improve the organic nitrogen fractions in a coconut plantation with acid soil

**DOI:** 10.1371/journal.pone.0277944

**Published:** 2023-03-10

**Authors:** Dongfen Huang, Xulin Liu, Hengfu Huan, Guodao Liu, An Hu

**Affiliations:** 1 Tropical Crops Genetic Resources Institute, Chinese Academy of Tropical Agricultural Sciences (CATAS) / Key Laboratory of Crop Gene Resources and Germplasm Enhancement in Southern China, Ministry of Agricultural and Rural Affairs, Haikou, Hainan, P.R. China; 2 College of Agronomy, Heilongjiang Bayi Agricultural University, Daqing, Heilingjiang, P.R. China; ICAR-National Rice Research Institute, INDIA

## Abstract

Intercropping green manure (GM) may be a good solution to the problems of acid soil in tropical plantations. Soil organic nitrogen (No) may change due to the application of GM. A three-year field experiment was conducted to determine the effect of different utilization patterns of *Stylosanthes guianensis* GM on soil No fractions in a coconut plantation. Three treatments were set: no GM intercropping (CK), intercropping and mulching utilization pattern (MUP), and intercropping and green manuring utilization pattern (GMUP). The content dynamics of soil total N (TN) and soil No fractions including of non-hydrolysable N (NHNo) and hydrolyzable N (HN) in the cultivated soil layer was examined. The results showed that after three years of intercropping, the TN content of the MUP and GMUP treatment was 29.4% and 58.1% respectively higher (*P* < 0.05) than those of the initial soil, and the No fractions content of GMUP and MUP treatment was 15.1%-60.0% and 32.7%-111.0% higher (*P* < 0.05) than those of the initial soil. The further results indicated that after three years of intercropping, compared with CK, GMUP and MUP could increase the content of TN by 32.6% and 61.7% respectively, and No fractions content was also increased by 15.2%-67.3% and 32.3%-120.3%% respectively (*P* < 0.05). The No fractions content of GMUP treatment was 10.3%-36.0% higher than those of MUP treatment (*P* < 0.05). These results indicated that intercropping *Stylosanthes guianensis* GM could significantly increase the soil N including of the TN an No fractions content, and the GMUP was more effective than MUP, therefore, GMUP is a better GM utilization pattern to improve the soil fertility and should be popularized in the tropical fruit plantation.

## Introduction

Coconut (*Cocos nucifera* L.) is the main crop in the tropics, especially in the Southeast Asia and South Pacific region. Coconut is also widely planted in the tropical China. In 2019, coconut, rubber (*Hevea brasiliensis*), and areca (*Areca catechu* L.) as the three main tropical crops in Hainan Province, which should be given priority to development [1]. The coconut industry is in the spotlight, but there are many problems including land degradation and coconut production (especially quality, which is decreasing) in Southeast Asia, especially China. Intercropping green manure (GM), especially leguminous GM, may be a good solution to these problems, as the addition of organic materials has a direct effect on soil organic matter (SOM) content and also ameliorates aluminum (Al) toxicity and reduces soil acidity, mainly by complexation, the liming effect, and N transformation [2–9]. Other impacts may include improved soil physical properties and microbial activity [10–13]. Stylosanthes (*Stylosanthes* sp.) is a leguminous GM commonly used in the tropics because it originates from tropics of the South Africa and can not survive the regions with the minimum temperature below 4°C. Among all Stylosanthes species, *Stylosanthes guianensis* is a perennial plant, suitable for acid soil, and is most widely planted in the tropics. As leguminous, intercropping with Stylosanthes may have a significant effect on soil N, which is essential for coconut growth. However, the effect of different forms of organic nitrogen (No) on tropical acid soil are variable, and No is the source and sink of inorganic N through mineralization [14]. Bremner [15] divided soil No into non-hydrolysable N (NHNo) and hydrolyzable N (HN), HN including hydrolyzable amino acid N (HAAN), hydrolyzable ammonium N (HAN), hydrolyzable amino sugar N (HASN), and hydrolyzable unidentified N (HUN). There are few studies on the effect of GM on soil No, and mainly focusing on the effect of chemical fertilizer N or the organic materials on soil No or the effect of GM on soil nitrate N, HAN, and other forms of inorganic N [16–20]. Xu et al. [21] found that most of the remaining N from manure applied with NPK was transferred into HASN in each size fraction and HAAN with a size fraction > 2 μm during the process of humification. Yu et al. [22] found that long-term application of Chinese milk vetch (*Astragalus sinicus* L.) GM under a rotation pattern could increase the soil total N (TN), dissolved total N, microbial biomass N, active N and improve soil fertility. Xie et al. [23] also found that Chinese milk vetch could significantly increase the HN and NHN contents in the soil. Huang et al. [24] found that long-term application of Chinese milk vetch could increase the proportion of soil HASN and HAAN. These studies have focused on the effects of annual or cross-year GM crop rotation on the soil No fractions, and the effects of intercropping perennial GM crops on the soil No fractions changes in tropical acid soils lack report.

Therefore, a three-year field trial was conducted to determine the effect of different GM utilization patterns on the changes in No fractions in tropical acid soil after Stylosanthes GM was intercropped in a coconut plantation.

## Materials and methods

### Green manures

*Stylosanthes guianensis* cv. Reyan No. 2 (a selection from CIAT184 which was registered as a new national variety by China Ministry of Agriculture) was used as GM crops. This is a perennial crop, and the growth point is about 20 cm, so the aboveground part taller than 25 cm was cut to be used as GM when the height was about 80 cm−150 cm at the vegetative stage.

### Experiment field

The experiment was conducted at the experimental station of the Chinese Academy of Tropical Agricultural Sciences in Danzhou, Hainan Province in China, which is located at the southern edge of the East Asian continental monsoon climate. This area has a humid tropical monsoon climate with plenty of sunshine and rain. The mean annual temperature is 23.5°C, the mean temperature in the hottest month is 27.8°C, the maximum temperature is 38°C, the mean temperature in the coldest month is 17.5°C, and the minimum temperature is 3.2°C. The soil is a Latosol with the following characteristics based on the soil analysis: pH 5.03, soil organic matter 1.06%, available N 31.5 mg kg^−1^, available P 6.45 mg kg^−1^, NH_4_AOc-extractable K 99.7 mg kg^−1^, HCl-extractable Fe 55.6 mg kg^−1^, HCl-extractable Mn 30.7 mg kg^−1^, HCl-extractable Cu 5.42 mg kg^−1^, and HCl-extractable Zn 4.97 mg kg^−1^, which means the soil is acidic, bears the low content of organic matter, and available nitrogen, but the soil bears the high content of mineral nutrients content in nature.

### Treatments

**Fig 1:**
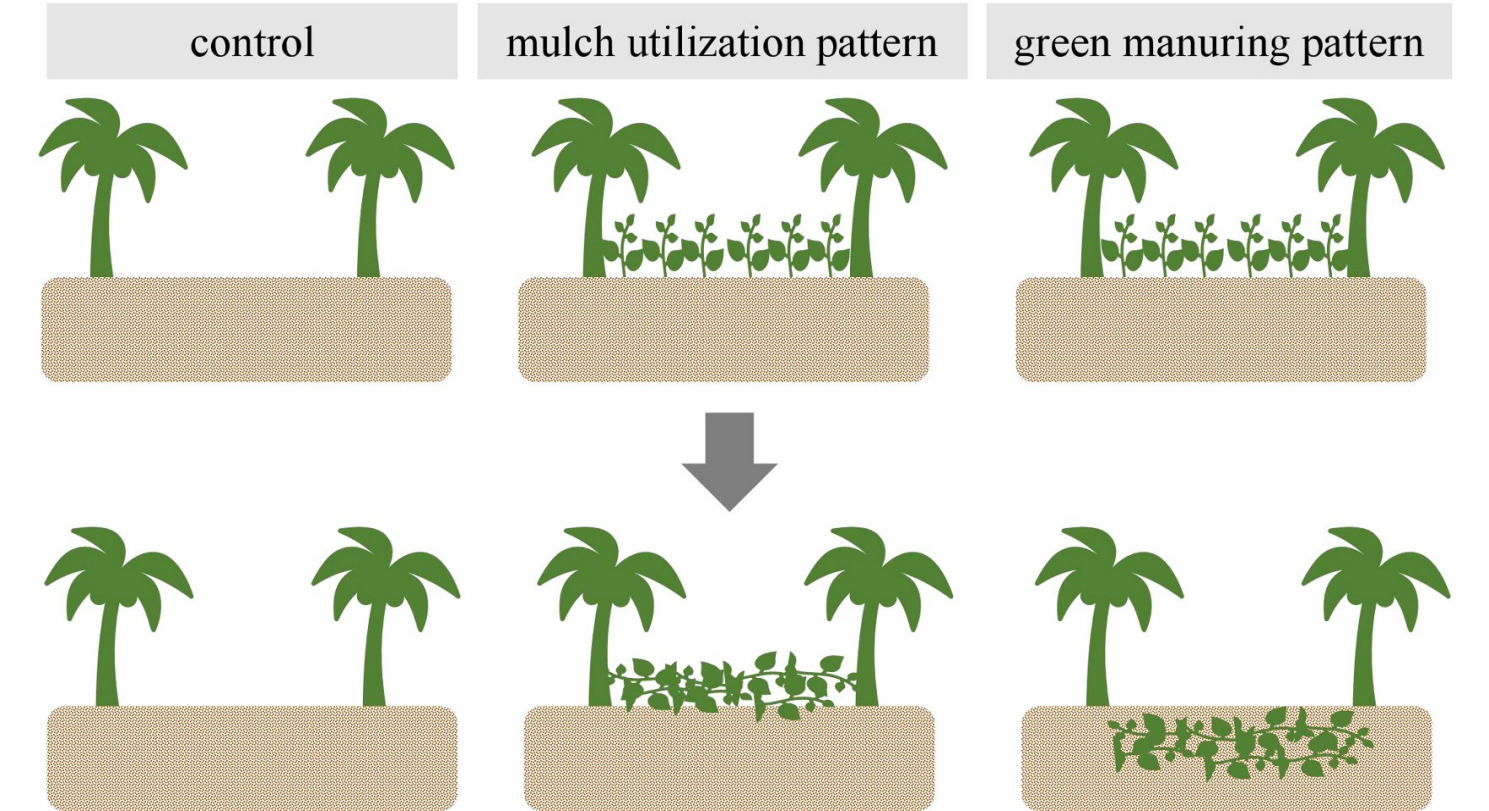
Pictorial treatment details of field image.

The study began in 2017 in newly planted coconut plantations. Three treatments were implemented based on a random block design (Fig 1): control (CK), mulch utilization pattern (MUP) and green manuring utilization pattern (GMUP). CK was not intercropped with Stylosanthes GM; the weeds were frequently cut by machine and left on the bare soil of the coconut tree rows. In MUP, intercropped GM was mulched around the coconut trees after GM was cut. In GMUP, intercropped GM was buried in a fertilization pit after GM was cut. Each plot had three rows of coconut trees, and each row had seven coconut trees; i.e., 21 coconut trees were planted. The row spacing was 6 m × 6 m, the plot area was 42 m × 18 m = 756 m^2^, and the total plot area was 6804 m^2^. The compound fertilizer (the nutrients content of N, P_2_O_5_, K_2_O is all 15% and totally is 45%) is applied and the rate is 45, 60 and 75 kg ha^-1^ respectively in the three years, the urea (N content is 46%) is applied at the rate of 30, 45, 60 kg ha^-1^ respectively in the three years, and the composted cattle manure (the content of C and N is 16.7% and 0.93% respectively) is applied at the rate of 2.25, 3.00, 4.50 t ha^-1^ respectively in the three years. The row spacing of Stylosanthes was 0.5 m × 0.5 m, and they were planted 0.5 m from the coconut trees to prevent the negative effects on coconut growth. No fertilizer was applied during the growth period of Stylosanthes, and the stubble height was 0.25 m. Stylosanthes and weed was cut twice in June and October every year. For the GM treatments, the biomass of Stylosanthes in the three years was averagely 14.9 t ha^-1^, and N content was 2.41%; for CK, the biomass of weed in the three years was averagely was 6.42 t ha^-1^ and N content was 1.52%. Soil samples were collected after three years Stylosanthes intercropping. The depth of the sample was 20 cm. Soil samples were brought to the laboratory where impurities were removed. Then, the soil was air-dried and passed through a 2-mm sieve, and then the soil samples were kept for analysis.

### Soil and plant analyses

The soil No fractions were determined by a sequential extraction procedure according to the acid hydrolysis–distillation method presented by Bremner [25]. A 5.00-g soil sample was weighed and placed in a hydrolysis tube. Two drops of n-octanol and 20 mL of 6 M HCI were added. The hydrolysis tube was then shaken to fully mix the soil and acid. The bottle was placed in a constant-temperature oven set to 110°C, and then the soil–acid mixture was hydrolyzed for 12 h. After hydrolysis, the sample was filtered through filter paper while it was still hot. The filtrate was collected in a 50 mL volumetric flask and cooled, and the residue was rinsed with a small amount of deionized water several times with a constant volume of 50 mL. Then, the pH was adjusted, 20 mL of the hydrolysate was placed into a 50-mL beaker, and the beaker with the filtrate was placed on crushed ice. A 5 M NaOH solution was slowly added drop by drop and then stirred until the pH of the hydrolysate reached about 5. The hydrolysate was neutralized with 0.5 M NaOH so that the pH of the hydrolysate reached 6.5 ± 0.3. Then, the acidic solution was transferred to a 50-mL volumetric bottle with a small funnel and the volume was kept to scale. (1) For the determination of THN: Before sampling, the volumetric flask was inverted several times to ensure that the suspension was uniform; a wide pipette was used to absorb 5 mL of the acidic solution that was then added to the boiling tube. After that, 0.5 g of nitrogenous mixture catalyst and 2 mL of concentrated sulfuric acid were added, boiled for 1.5 h, and then left to cool. The N content in the extract was analyzed by a Kjeldahl N meter. (2) For the determination of HAN: Neutralized acid hydrolysate (20 mL) was placed in a dissolving tube and 0.14 g MgO was added. Distillation was conducted for about 2 min, and then the titration was performed. (3) For the determination of HAN and HASN: Neutralized acid hydrolysate (20 mL) was placed in a boiling tube, and 20-mL monoboric acid buffer solution (pH = 11.2) was added and distilled for 4 min, followed by titration. The determination results minus the ASNo content in (2) represent the ASNo content. (4) For the determination of HAAN: First, 5 mL of neutralized acid hydrolysate was placed in a 10 mL test tube, l mL of 0.5 M NaOH solution was added, and then the solution was heated in boiling water until it was reduced to 2–3 mL. The solution was removed and allowed to cool. Following this, 0.5 g of citric acid and 0.1 g of ninhydrin were added, and we placed the test tube in a boiling water bath. After heating for 1 min, the solution was shaken for 10 s, and then it was placed back in the water bath for 9 min. After cooling, 10 mL of monoboric acid phosphate buffer and 1 mL of 5 M NaOH were added and distilled for 4 min. (5) HUN = THN—(HAN + HASN + HAAN). Total N (TN) and organic N fractions were measured by the micro-Kjeldahl method presented by Bremner [25]. The non-hydrolyzable N (acid-insoluble N) was estimated by subtracting the total hydrolyzable N from TN according to the method presented by Bremner [25]. Soil pH was measured using a 1:2.5 soil/water suspension and a thin, combined glass/calomel electrode pH meter (Model pp-15, Sartorius AG, Göttingen, Germany) according to the method outlined by Lu [26]. The bulk density of the soil was determined using the cutting-ring method of Lu [26]. Soil organic matter was analyzed using the wet combustion method as described by Lu [26]. Soil available phosphorus (P) was extracted using 0.03 M NH_4_F with 0.025 M HCl according to the method described by Bray and Kurtz [27]. The P concentrations in the extracts were determined by a spectrophotometer (Model V-1200, Shanghai Mapada Instrument Co., Ltd.) using the molybdenum-blue method of Lu [26]. Soil available K was extracted using 1 M NH_4_OAc (pH 7) according to the method of Wood and Del Turk [28], and K concentrations in the extracts were analyzed using a flame photometer [26]. GM samples were digested with HNO_3_ using a microwave digestion system (CEM MARS, Matthews, NC, USA), and the P concentrations in the solution were determined by a spectrophotometer (Model V-1200; Shanghai Mapada Instrument Co., Ltd.) using the molybdenum-blue method described by Lu [26].

### Statistical analysis

All results were reported as means ± standard deviation (SD) for three replicates. Two-way of variance (ANOVA) and Duncan’s multiple range test were performed to determine the differences among the GM utilization pattern treatments in terms of the TN and No fractions content means of the three years, and paired comparisons t-test was performed to determine the TN and No fractions content differences between the initial soil and content means of the three years intercropping for the same treatment. All statistical analyses were performed using the SAS software package (version 9.4) (SAS Institute Inc., Cary, NC, USA) [29]. A difference at *P* < 0.05 level was considered as statistically significant. The tables were produced using Microsoft Office Word 2010.

## Results

### Soil total nitrogen

The results from Table 1 indicate that after three years intercropping, the TN content in the MUP and GMUP treatment increased respectively by 29.4% and 58.1% (*P* < 0.05) than that of the initial soil, however, the TN content in CK decreased 3.16% (*P* < 0.05) than that of the initial soil. The result also indicated that during the three years of intercropping, the TN content of the MUP and GMUP treatments was higher than that of CK (*P* < 0.05) by 32.6% and 61.7%, respectively, and the TN content in the GMUP treatment was 22.0% higher than MUP treatment (*P* < 0.05). These results indicated that MUP and GMUP treatment all had a positive effect on the soil TN content increase, but GMUP was more effective in increasing the TN content than GMU, which means GMUP had the better effect in increasing the TN content than MUP.

**Table 1:** Total nitrogen content of different treatments in the initial soil and the soil after different intercropping years (mg·kg^-1^).

Treatments	Initial soil	Year after intercropping			
		1	2	3	Mean
CK	722.7±1.92aA	710.2±4.97	700.0±2.29	689.3±4.76	699.8±4.49aB
MUP	717.2±6.30aB	791.5±2.36	921.8±6.77	1069.8±12.74	927.7±49.5bA
GMUP	715.7±3.00aB	928.3±3.56	1115.3±7.10	1351.2±2.40	1131.6±75cA

CK: without intercropping with Stylosanthes GM; the weeds were frequently cut by machine and left on the bare soil of the coconut tree rows; MUP: intercropped GM was mulched around the coconut trees after the GM was cut; GMUP: intercropped GM was buried in a fertilization pit after the GM was cut.

The value is the mean±SE (n = 3); The value with the same lowercase letters in the same column are not significantly different at the 0.05 level for the treatments in the same year; The value with the same capital letters in the same row are not significantly different at the 0.05 level for the treatments in the different years.

### Total hydrolyzable nitrogen

The total hydrolyzable nitrogen (THN) is an important component of soil No. GM intercropping and application probably affected THN due to the high content of nitrogen in leguminous GM. The results (Table 2) showed that after three years intercropping with GM, the THN content in the MUP and GMUP treatment increased significantly by 36.6% and 71.1% (*P* < 0.05) respectively compared with the initial soil, however, the THN content in CK decreased 3.31% (*P* < 0.05) than that of the initial soil. These results also indicated that during the three years intercropping with GM, the THN content in the MUP and GMP treatments was 41.6% and 77.2% higher, respectively, than that of CK (*P* < 0.05); and the value in the GMUP treatment was 25.2% higher than MUP treatment (*P* < 0.05). These results indicated that MUP and GMUP treatment all had a positive effect on the soil THN content increase, but GMUP was more effective in increasing the THN content than MUP.

**Table 2:** Total hydrolyzable nitrogen fractions content of different treatments in the initial soil and the soil after three intercropping years (mg·kg^-1^).

Treatments	Initial soil	Year after intercropping			
		1	2	3	Mean
CK	473.5±2.75aA	467.1±2.07	460.8±3.35	445.5±2.31	457.8±4.25cB
MUP	474.3±1.92aB	541.5±2.75	641.0±1.61	761.9±5.73	648.1±39.1bA
GMUP	474.3±4.21aB	652.2±5.83	795.0±3.62	987.2±3.66	811.4±59.5aA

CK: without intercropping with Stylosanthes GM; the weeds were frequently cut by machine and left on the bare soil of the coconut tree rows; MUP: intercropped GM was mulched around the coconut trees after the GM was cut; GMUP: intercropped GM was buried in a fertilization pit after the GM was cut.

The value is the mean±SE (n = 3); The value with the same lowercase letters in the same column are not significantly different at the 0.05 level for the treatments in the same year; The value with the same capital letters in the same row are not significantly different at the 0.05 level for the treatments in the different years.

### Hydrolyzable nitrogen fractions

The contents of hydrolysable N fractions in the soil were affected by the MUP and GMUP treatments (Table 3). The results indicated that after three years intercropping, the hydrolyzable ammonia N (HAN) content in the MUP and GMUP treatment increased significantly by 43.3% and 66.7% (*P* < 0.05) respectively compared with the initial soil, however, the HAN content in CK decreased by 9.62% (*P* < 0.05) than that of the initial soil. These results also indicated that during the three years intercropping with GM, the HAN content in the MUP and GMUP treatments was 57.2% and 73.3% higher, respectively, than that of CK (*P* < 0.05); and the value in the GMUP treatment was 10.3% higher than MUP treatment (*P* < 0.05). These results indicated that MUP and GMUP treatment all had a positive effect on the soil HAN content increase, but GMUP was more effective in increasing the HAN content than MUP.

**Table 3:** Organic nitrogen fractions content of different treatments in the initial soil and the soil after three intercropping years (mg·kg^-1^).

No fractions	Treatments	Initial soil	Year after intercropping			
			1	2	3	Mean
HAN	CK	159.4±2.20aA	152.1±3.36	142.8±4.87	137.2±1.71	144.0±3.43cA
	MUP	158.0±3.27aB	171.1±4.02	235.9±1.19	272.1±2.22	226.4±18.2bA
	GMUP	149.8±6.29aB	190.3±5.59	250.8±3.11	307.8±8.46	249.6±21.1aA
HAAN	CK	191.0±2.35aA	185.1±2.33	184.3±2.14	180.6±2.95	183.4±1.75cA
	MUP	180.6±0.94aB	190.3±3.67	202.2±5.10	241.0±3.94	211.2±9.74bA
	GMUP	188.5±0.78aB	230.4±4.04	284.8±2.91	346.1±3.54	287.1±20.6aA
HASN	CK	22.7±0.76aA	22.1±0.33	20.2±0.70	19.5±0.14	20.6±0.54cB
	MUP	20.9±0.43aB	23.9±0.20	26.7±0.40	30.1±0.22	26.9±1.10bA
	GMUP	21.3±0.59aB	26.5±0.57	32.5±0.61	39.5±0.77	32.8±2.30aA
HUN	CK	100.4±3.42aA	107.8±0.87	113.5±7.37	108.1±1.78	109.8±2.92cB
	MUP	114.8±4.83aB	156.2±2.29	176.2±6.93	218.7±6.22	183.7±11.8bA
	GMUP	114.7±2.98aB	204.9±0.97	226.9±2.98	293.8±6.20	241.9±16.6aA

CK: without intercropping with Stylosanthes GM; the weeds were frequently cut by machine and left on the bare soil of the coconut tree rows; MUP: intercropped GM was mulched around the coconut trees after the GM was cut; GMUP: intercropped GM was buried in a fertilization pit after the GM was cut.

No: organic nitrogen; HAN: hydrolyzable ammonia nitrogen; HAAN: hydrolyzable amino acid nitrogen; HASN: hydrolyzable amino sugar nitrogen; HUN: hydrolyzable unknown nitrogen.

The value is the mean±SE (n = 3); The value with the same lowercase letters in the same column are not significantly different at the 0.05 level for the treatments in the same year; The value with the same capital letters in the same row are not significantly different at the 0.05 level for the treatments in the different years.

The HAAN content was highest among the No fractions in this research, therefore, the change of HAAN content would produce the important effect on the No. The result indicated that after three years of intercropping (Table 3), the HAAN content in the MUP and GMUP treatment increased significantly by 16.9% and 52.3% (*P* < 0.05) respectively compared with the initial soil, however, the HAAN content in CK didn’t have the difference (*P* > 0.05) with the initial soil. The results also indicated that the HAAN content in the MUP and GMP treatments was 15.2% and 56.6% higher, respectively, than that of CK (*P* < 0.05); and the value in the GMUP treatment was 36.0% higher than MUP treatment (*P* < 0.05). These results indicated that MUP and GMUP treatment all had a positive effect on the soil HAAN content increase, but GMUP was more effective in increasing the HAAN content than MUP.

The HASN content was low in the No fractions, and the HASN content of the two treatments increased significantly (*P* < 0.05) every year (Table 3). After three years intercropping with GM, the HASN content in the MUP and GMUP treatment increased significantly by 28.7% and 53.8% (*P* < 0.05) respectively compared with the initial soil, however, the HASN content in CK hadn’t the difference (*P* > 0.05) with the initial soil. These results also indicated that during the three years intercropping with GM, the HASN content in the MUP and GMP treatments was 30.6% and 59.4% higher, respectively, than that of CK (*P* < 0.05); and the value in the GMUP treatment was 22.1% higher than MUP treatment (*P* < 0.05). These results indicated that MUP and GMUP treatment all had a positive effect on the soil HASN content increase, but GMUP was more effective in increasing the HASN content than MUP.

The results from Table 3 indicated that after three years intercropping with GM, the HUN content in the MUP and GMUP treatment increased significantly by 60.0% and 111.0% (*P* < 0.05) respectively compared with the initial soil, the HUN content in CK also increased 9.42% (*P* < 0.05) than that of the initial soil. The results also showed that during the three years intercropping with GM, the HUN content in the MUP and GMP treatments was 67.3% and 120.3% higher, respectively, than that of CK (*P* < 0.05); and the value in the GMUP treatment was 31.7% higher than MUP treatment (*P* < 0.05). These results indicated that MUP, GMUP and CK all had a positive effect on the soil HUN content increase after three years intercropping, but GMUP was more effective in increasing the HUN content than MUP.

### Non-hydrolyzable nitrogen

The results from Table 4 showed that after three years intercropping with GM, the NHN content in the MUP and GMUP treatment increased significantly by 15.1% and 32.7% (*P* < 0.05) respectively compared with the initial value, however, the NHN content in CK decreased 2.87% (*P* < 0.05) than that of the initial soil. The results from also indicated that during the three years intercropping with GM, the NHN content in the MUP and GMP treatments was 15.5% and 32.3% higher, respectively, than that of CK (*P* < 0.05); and the value in the GMUP treatment was 14.5% higher than MUP treatment (*P* < 0.05). These results indicated that MUP and GMUP treatment all had a positive effect on the soil NHN content increase, and GMUP was more effective in increasing the HASN content than GMU.

**Table 4:** Non-hydrolyzable nitrogen content of different treatments in the initial soil and the soil after three intercropping years (mg·kg^-1^).

Treatments	Initial soil	Year after intercropping			
		1	2	3	Mean
CK	249.2±4.41aA	243.1±2.90	239.2±3.42	243.8±3.56	242.0±2.21cB
MUP	242.8±7.51aB	250.0±1.89	280.8±7.22	307.9±18.4	279.6±12.4bA
GMUP	241.3±2.62aB	276.2±8.91	320.3±3.56	364.0±4.54	320.2±16.0aA

CK: without intercropping with Stylosanthes GM; the weeds were frequently cut by machine and left on the bare soil of the coconut tree rows. MUP: intercropped GM was mulched around the coconut trees after the GM was cut; GMUP: intercropped GM was buried in a fertilization pit after the GM was cut.

The value is the mean±SE (n = 3); The value with the same lowercase letters in the same column are not significantly different at the 0.05 level for the treatments in the same year; The value with the same capital letters in the same row are not significantly different at the 0.05 level for the treatments in the different years.

### The contribution of organic nitrogen fractions to the total nitrogen change

**Fig 2:**
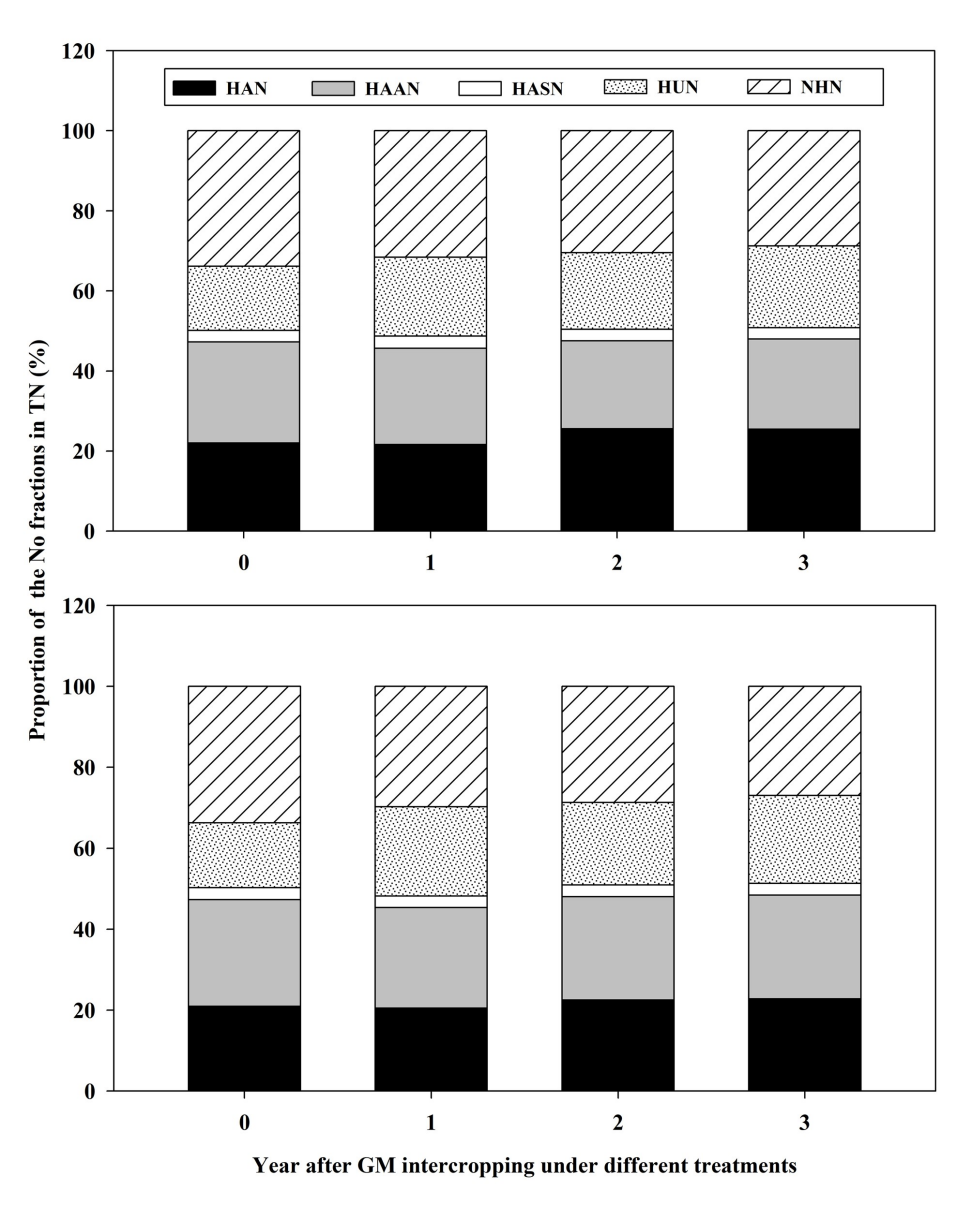
Proportion of organic nitrogen to total nitrogen for each soil fraction. The left figure is the MUP treatment: intercropped GM was mulched around the coconut trees after the GM was cut; The right figure is the GMUP treatment: intercropped GM was buried in a fertilization pit after the GM was cut.

After three years of intercropping, the proportion of No fractions to TN was NHN > HAN > HAAN > HUN > HASN in the MUP treatment (Fig 2). Among them, the proportion of HUN and HAN in TN increased, and the annual increase was averagely 3.76% and 2.20% respectively during the three years intercropping; The HASN had the smallest proportion of TN; it was relatively stable at 3.0% or so during the three years intercropping. The proportion of NHN and HAAN in TN decreased, and the annual decrease was averagely 3.58% and 2.35% respectively during the three years intercropping. In the GMUP treatment, the proportion of No in TN was NHN > HAAN > HAN > HUN > HASN after three years of intercropping. The proportion of NHN, HUN and HAN increased, and the annual increase was averagely 5.25%, 5.37% and 0.99% respectively during the three years intercropping. The proportion of the other fractions to TN was relatively stable.

These results indicated that the proportion of HAAN, HASN and HUN to TN in the GMUP treatment was higher than that in the MUP treatment during the three years intercropping, but the proportion of HAN and UHN to TN was higher in the MUP treatment than that in the GMUP treatment.

## Discussion

Nitrogen is an essential element and is often deficient in tropical acid soil. The application of synthetic N fertilizer is a common measure to solve this deficiency problem. However, long-term chemical N application results in numerous problems including land degradation, decreased quality of agricultural products and yield, as well as environmental pollution [30]. Of late, a reduction in chemical fertilizer application, especially N fertilizer, has become more acceptable for solving these problems. Among the many possible measures, the application of organic manure, including GM and especially leguminous GM, is one of the best choices, and GM can be used to import N into soil and replace chemical N fertilizer. It is one of the most effective measures to improve soil nitrogen level and expand the soil N pool [31–33]. Coconut and the other fruit are commonly planted in the mountain or slope land in China and the other southeast Asia, under this condition, the fertilizer especially the organic manure is difficult to be applied due to the poor traffic condition, intercropping of GM especially the leguminous GM is a good way to solve the problems. After the application of leguminous GM, the acid soil No fractions, will be probably changed because GM contains high amounts of N and organic matter that will affect the N content and transformation. However, the research on the No fractions changes of different utilization patterns after GM intercropping lack of report. Therefore, in order to illustrate this, a three-year field experiment on changes in the No fractions under the different utilization patterns after intercropping with leguminous GM was conducted, and the results indicated that that the content of TN, NHN and THN including different fractions (HAN, HAAN, HASN and HUN), increased significantly in the two treatments (especially in the GMUP treatment) compared with CK. This means that the application of GM had a significantly positive effect on soil N accumulation. The increase was obviously because the GM intercropping and application could bring more N (averagely 359.1 kg/ha) than CK (averagely 97.6 kg/ha). The result is consistent with other findings on the effect of organic materials, including the effects of different utilization patterns of GM on the soil No fractions, but it is also inconsistent with some studies, especially with respect to the different N fractions [21–24, 34]. Xu et al. [21] found that a pig manure + NPK treatment could increase the content of HASN and HAAN. The research results from Yu et al. [22] indicated that mung bean [*Vigna radiata* (L.) Wilczek], a leguminous GM, could increase the soil organic N pool. Xie et al. [23] showed that a mixed application of N fertilizer with Chinese milk vetch (*Astragalus sinicus* L.) GM resulted in significantly higher values of N in all forms with the exception of HUN. The application rate can affect the content and proportion of the soil No fractions. Wu et al. [35] found that compared with CK (no corn residue application), the 33% corn residue application treatment showed a significantly higher concentration of soil hydrolyzable NH_4_^+^-N, HAAN, and HASN in both the 0–10- and 10–20-cm soil layers, whereas the 67% corn residue application treatment and the 100% corn residue application treatment significantly increased the concentration and proportion of soil HUN but decreased the HAN, HAAN, and HASN proportions in the 10–20 cm soil layer. Huang et al. [24] reported that long-term application of Chinese milk vetch as GM with or without chemical fertilizer could increase all soil No fractions including the HAN, HAAN, HASN, HUN, NHN, and even TN. These results indicated that the application of organic manure, including GM mixed with or without phosphate fertilizer, could increase the different soil No fractions, but the increase depended on the type of organic manure fraction, as well as the GM and the organic manure application rate, the timing, the type, and whether it was mixed with chemical fertilizer.

The GM application increased the No fraction was not only mainly due to the large amount of N with different forms in GM, but also the soil microbes play important roles in the turnover of different fractions of soil No, even the turnover of No and inorganic N, because GM can effectively change the microbial biomass, which has a positive and significant effect on soil N hydrolase, and microbial N could actively regulate the transformation of No through enzymatic mineralization [36]. On another hand, the increase in No may be due to the effect of long-term GM application on soil microorganisms. It has been reported that the soil microbial biomass increased, the structure of the biological population improved, and the content of HAN increased by changing the transformation of No fractions [34]. HAAN is the main source of N from soil [37], and together with HAN, is the main component of soil available N and is an important index of soil N supply potential in agricultural planting [38]. Our research found that the content of HAAN in MUP and GMUP treatment increased by 33.4% and 91.6% respectively after three years of intercropping with GM. HASN is a residual substance from the cell wall of soil microorganisms that occupies a low proportion of HN, is more stable in soil, and can supply crops after mineralization [39]. Our results indicated that the content of HASN increased by 54.1% and 102.1% in the GMUP and MUP treatments, respectively. Xie et al. [40] found that Chinese Milk Vetch GM rotation increased the number of soil fungi, nitrogen-fixing bacteria, and actinomycetes, and also optimized the soil microbial community. After the death of these microorganisms, their cell walls remained in the soil, which increases the accumulation of HASN in soil [41]. The influence of GM on HASN may be mainly due to its influence on the soil microbial structure and the increase in microbial biomass. After a microorganism dies, the residues in its cell wall enter the soil, increasing the accumulation of HASN in soil. HUN is one of the main sources of soil reactive N. In this study, the highest increase of HUN was 102.2% and 171.7% in the GMUP and MUP treatments. The highest increase occurred in the third year after intercropping. With the increase in intercropping duration, the effect on the HUN content in soil was more significant. HUN is not mineralized easily, and its components are generally α-amino acid N, aliphatic amines, and aromatic amines, which are relatively less biologically effective because of their comparatively slow mineralization rate, resulting in rapid accumulation in the soil [41]. In addition to the accumulation of N by GM itself, the increase in the content of HUN may be due to the increase in microbial activity, which increases the growth and metabolic rates and the content of HUN in soil. NHN is not easily mineralized, resulting in its accumulation in the soil. Our result indicated that the content of UHN increased by 26.3% and 49.3% after intercropping GM in the MUP and GMUP treatment, a result similar to previous research. The content of NHN was higher than that of other No fractions; compared with CK, the content of NHN increased less than that of other N components, which indicated that the stability of NHN in soil was higher, which is consistent with the results of Lü et al. [42]. NHN is one of the main forms that accumulate in response to increasing amounts of N under GM application; the result of this research was different from this and was probably related to the soil texture and the GM used. After intercropping of *Stylosanthes* GM in the tropics, the No content increased, indicating that GM intercropping under high temperature and rainy weather in a Latosol with a low N level is an effective method to increase the soil No content. Wu et al. [38] found that the application of 2.5 t ha^−1^ GM could significantly increase the contents of HAN, HASN, and soluble No and had a good effect on increasing the supply of active soluble No; however, 5.0 t ha^−1^ and 7.5 t ha^−1^ GM increased the contents of HUN and stable No, respectively.

The proportion of THN and HUN to TN in the GMUP and MUP treatments increased after intercropping with Stylosanthes GM, and the proportion of UHN to TN decreased. In contrast to the MUP treatment, the GMUP treatment reduced the proportion of HAAN in TN. Huang et al. [24] found that the application of GM could increase the proportion of ASN and AAN in TN, and decrease the proportion of AN in TN.

MUP and GMUP are the two most commonly used GM utilization patterns in tropical fruit and economic forest plantation. In the comparison of different utilization methods of intercropping GM, the GMUP was more effective than MUP. The reason was probably that the water content of crop residues will decrease after mulching, and it is more difficult to ensure contact with soil microorganisms and water compared with GM; the decomposition rate of organic matter residue was lower than that under GM treatment, and the Stylosanthes residue on the soil surface was affected by erosion caused by rain water, while the N loss was restricted under the condition of green manuring [43].

In conclusion, intercropping of GM with two different utilization pattern all could increase the coconut plantation soil TN and No fractions, but the GMUP had more significant effect than MUP, and was a good GM utilization pattern for the tropical fruit plantation.

## Supporting information

S1 Fig Proportion of organic nitrogen to total nitrogen for each soil fraction. The top figure is the MUP treatment: intercropped GM was mulched around the coconut trees after the GM was cut; The bottom figure is the GMUP treatment: intercropped GM was buried in a fertilization pit after the GM was cut. HAN: hydrolyzable ammonia nitrogen; HAAN: hydrolyzable amino acid nitrogen; HASN: hydrolyzable amino sugar nitrogen; HUN: hydrolyzable unknown nitrogen.
(PDF)

S1 Table Total nitrogen content of different treatments in the initial soil and the soil after different intercropping years (mg·kg^-1^). CK: without intercropping with Stylosanthes GM; the weeds were frequently cut by machine and left on the bare soil of the coconut tree rows; MUP: intercropped GM was mulched around the coconut trees after the GM was cut; GMUP: intercropped GM was buried in a fertilization pit after the GM was cut. The value is the mean±SE (n = 3); The value with the same lowercase letters in the same column are not significantly different at the 0.05 level for the treatments in the same year; The value with the same capital letters in the same row are not significantly different at the 0.05 level for the treatments in the different years.
(PDF)

S2 Table Total hydrolyzable nitrogen fractions content of different treatments in the initial soil and the soil after three intercropping years (mg·kg^-1^). CK: without intercropping with Stylosanthes GM; the weeds were frequently cut by machine and left on the bare soil of the coconut tree rows; MUP: intercropped GM was mulched around the coconut trees after the GM was cut; GMUP: intercropped GM was buried in a fertilization pit after the GM was cut. The value is the mean±SE (n = 3); The value with the same lowercase letters in the same column are not significantly different at the 0.05 level for the treatments in the same year; The value with the same capital letters in the same row are not significantly different at the 0.05 level for the treatments in the different years.
(PDF)

S3 Table Organic nitrogen fractions content of different treatments in the initial soil and the soil after three intercropping years (mg·kg^-1^). CK: without intercropping with Stylosanthes GM; the weeds were frequently cut by machine and left on the bare soil of the coconut tree rows; MUP: intercropped GM was mulched around the coconut trees after the GM was cut; GMUP: intercropped GM was buried in a fertilization pit after the GM was cut. No: organic nitrogen; HAN: hydrolyzable ammonia nitrogen; HAAN: hydrolyzable amino acid nitrogen; HASN: hydrolyzable amino sugar nitrogen; HUN: hydrolyzable unknown nitrogen. The value is the mean±SE (n = 3); The value with the same lowercase letters in the same column are not significantly different at the 0.05 level for the treatments in the same year; The value with the same capital letters in the same row are not significantly different at the 0.05 level for the treatments in the different years.
(PDF)

S4 Table Non-hydrolyzable nitrogen content of different treatments in the initial soil and the soil after three intercropping years (mg·kg^-1^). CK: without intercropping with Stylosanthes GM; the weeds were frequently cut by machine and left on the bare soil of the coconut tree rows. MUP: intercropped GM was mulched around the coconut trees after the GM was cut; GMUP: intercropped GM was buried in a fertilization pit after the GM was cut. The value is the mean±SE (n = 3); The value with the same lowercase letters in the same column are not significantly different at the 0.05 level for the treatments in the same year; The value with the same capital letters in the same row are not significantly different at the 0.05 level for the treatments in the different years.
(PDF)
