# Supplementary material for: Intercropping of Stylosanthes green manure could improve the organic nitrogen fractions in a coconut plantation with acid soil
Source: PLoS One. 2023 Mar 10;18(3):e0277944. doi: 10.1371/journal.pone.0277944 (PMC10004503; doi:10.1371/journal.pone.0277944)
Supplement: S1 Table — CK: without intercropping with Stylosanthes GM; the weeds were frequently cut by machine and left on the bare soil of the coconut tree rows; MUP: intercropped GM was mulched around the coconut trees after the GM was cut; GMUP: intercropped GM was buried in a fertilization pit after the GM was cut. The value is the mean±SE (n = 3); The value with the same lowercase letters in the same column are not significantly different at the 0.05 level for the treatments in the same year; The value with the same capital letters in the same row are not significantly different at the 0.05 level for the treatments in the different years. (PDF) [file pone.0277944.s002.pdf]

S1 Table. Total nitrogen content of different treatments in the initial soil and the soil after different intercropping years ($mg \cdot kg^{-1}$).

Treatments	Replication	Initial soil	Year after intercropping		
			1	2	3
CK	1	725.5	713.0	701.5	687.0
	2	723.5	717.0	695.5	682.5
	3	719.0	700.5	703.0	698.5
MUP	1	715.0	790.5	909.0	1052.0
	2	729.0	796.0	924.5	1063.0
	3	707.5	788.0	932.0	1094.5
GMUP	1	721.5	933.5	1102.5	1346.5
	2	711.5	930.0	1127.0	1354.5
	3	714.0	921.5	1116.5	1352.5
