# Supplementary material for: Intercropping of Stylosanthes green manure could improve the organic nitrogen fractions in a coconut plantation with acid soil
Source: PLoS One. 2023 Mar 10;18(3):e0277944. doi: 10.1371/journal.pone.0277944 (PMC10004503; doi:10.1371/journal.pone.0277944)
Supplement: S2 Table — CK: without intercropping with Stylosanthes GM; the weeds were frequently cut by machine and left on the bare soil of the coconut tree rows; MUP: intercropped GM was mulched around the coconut trees after the GM was cut; GMUP: intercropped GM was buried in a fertilization pit after the GM was cut. The value is the mean±SE (n = 3); The value with the same lowercase letters in the same column are not significantly different at the 0.05 level for the treatments in the same year; The value with the same capital letters in the same row are not significantly different at the 0.05 level for the treatments in the different years. (PDF) [file pone.0277944.s003.pdf]

S2 Table. Total hydrolyzable nitrogen fractions content of different treatments in the initial soil and the soil after three intercropping years (mg·kg^{-1}).

Treatments	Replication	Initial soil	Year after intercropping		
			1	2	3
CK	1	468.0	468.5	455.5	441.5
	2	476.0	469.8	460.0	445.5
	3	476.5	463.0	467.0	449.5
MUP	1	471.5	537.0	640.5	771.3
	2	473.5	546.5	644.0	763.0
	3	478.0	541.0	638.5	751.5
GMUP	1	479.5	650.5	789.0	986.0
	2	466.0	643.0	801.5	981.5
	3	477.5	663.0	794.5	994.0
