# Supplementary material for: Intercropping of Stylosanthes green manure could improve the organic nitrogen fractions in a coconut plantation with acid soil
Source: PLoS One. 2023 Mar 10;18(3):e0277944. doi: 10.1371/journal.pone.0277944 (PMC10004503; doi:10.1371/journal.pone.0277944)
Supplement: S3 Table — CK: without intercropping with Stylosanthes GM; the weeds were frequently cut by machine and left on the bare soil of the coconut tree rows; MUP: intercropped GM was mulched around the coconut trees after the GM was cut; GMUP: intercropped GM was buried in a fertilization pit after the GM was cut. No: organic nitrogen; HAN: hydrolyzable ammonia nitrogen; HAAN: hydrolyzable amino acid nitrogen; HASN: hydrolyzable amino sugar nitrogen; HUN: hydrolyzable unknown nitrogen. The value is the mean±SE (n = 3); The value with the same lowercase letters in the same column are not significantly different at the 0.05 level for the treatments in the same year; The value with the same capital letters in the same row are not significantly different at the 0.05 level for the treatments in the different years. (PDF) [file pone.0277944.s004.pdf]

S3 Table. Organic nitrogen fractions content of different treatments in the initial soil and the soil after three intercropping years (mg·kg^{-1}).

No fractions	Treatments	Replication	Initial soil	Year after intercropping		
				1	2	3
HAN	CK	1	155.6	158.1	148.3	135.8
		2	163.3	151.6	147.1	135.3
		3	159.3	146.5	133.1	140.6
	MUP	1	161.9	163.1	233.6	269.3
		2	160.6	175.8	236.5	270.6
		3	151.5	174.5	237.6	276.5
	GMUP	1	158.6	198.8	257.0	319.9
		2	137.6	179.8	248.0	291.5
		3	153.1	192.5	247.4	312.0
HAAN	CK	1	195.3	180.5	180.1	174.8
		2	190.8	187.0	187.1	183.1
		3	187.1	187.9	185.8	184.0
	MUP	1	180.6	192.9	207.4	240.6
		2	179.0	194.9	192.0	248.0
		3	182.3	183.0	207.3	234.4
	GMUP	1	189.5	223.3	279.0	342.0
		2	187.0	230.8	288.1	343.1
		3	189.1	237.3	287.3	353.1
HASN	CK	1	21.3	22.7	19.1	19.7
		2	23.9	21.6	21.5	19.3
		3	23.1	21.9	20.1	19.7
	MUP	1	21.0	24.3	26.8	30.5
		2	21.6	23.9	25.9	29.7
		3	20.1	23.6	27.3	30.1
	GMUP	1	22.1	25.4	31.5	38.8
		2	21.8	27.3	33.6	41.0
		3	20.2	26.8	32.5	38.6
HUN	CK	1	95.9	107.2	108.1	111.4
		2	98.2	109.6	104.3	107.9
		3	107.1	106.7	128.1	105.2
	MUP	1	108.0	156.7	172.7	230.9
		2	112.3	152.0	189.6	214.7
		3	124.2	159.9	166.4	210.5
	GMUP	1	109.3	203.1	221.5	285.3
		2	119.6	205.3	231.8	305.9
		3	115.1	206.4	227.4	290.3
