# Supplementary material for: Intercropping of Stylosanthes green manure could improve the organic nitrogen fractions in a coconut plantation with acid soil
Source: PLoS One. 2023 Mar 10;18(3):e0277944. doi: 10.1371/journal.pone.0277944 (PMC10004503; doi:10.1371/journal.pone.0277944)
Supplement: S4 Table — CK: without intercropping with Stylosanthes GM; the weeds were frequently cut by machine and left on the bare soil of the coconut tree rows. MUP: intercropped GM was mulched around the coconut trees after the GM was cut; GMUP: intercropped GM was buried in a fertilization pit after the GM was cut. The value is the mean±SE (n = 3); The value with the same lowercase letters in the same column are not significantly different at the 0.05 level for the treatments in the same year; The value with the same capital letters in the same row are not significantly different at the 0.05 level for the treatments in the different years. (PDF) [file pone.0277944.s005.pdf]

S4 Table. Non-hydrolyzable nitrogen content of different treatments in the initial soil and the soil after three intercropping years (mg·kg^{-1}).

Treatments	Replication	Initial soil	Year after intercropping		
			1	2	3
CK	1	257.5	244.5	246.0	245.5
	2	247.5	247.3	235.5	237.0
	3	242.5	237.5	236.0	249.0
MUP	1	243.5	253.5	268.5	280.8
	2	255.5	249.5	280.5	300.0
	3	229.5	247.0	293.5	343.0
GMUP	1	242.0	283.0	313.5	360.5
	2	245.5	287.0	325.5	373.0
	3	236.5	258.5	322.0	358.5
